# Differential Associations of Occupational and Total Physical Activity with Sleep Quality Among Adults in the United Arab Emirates: A Cross-Sectional Study

**DOI:** 10.3390/ijerph23070935

**Published:** 2026-07-21

**Authors:** Yousef Aljawarneh, Ahmad M. Al-Bashaireh, Saed Azizeh, Sarah Sanad, Shahad R. Alhefeiti, Maryam Alkaabi, Salaimah S. Aldhanhani, Shama A. Aldhanhani, Haleimah Alkaabi, Aisha Alkaabi, Hana Ghunaim

**Affiliations:** 1 Nursing Program, Health Science Division, Higher Colleges of Technology, Abu Dhabi P.O. Box 25026, United Arab Emirates; 2 Faculty of Nursing, Philadelphia University, Jarash Road, Amman P.O. Box 19392, Jordan; aalbashaireh@gmail.com; 3 Nursing Program, Health Science Division, Higher Colleges of Technology, Fujairah Campus, Fujairah P.O. Box 25026, United Arab Emirates; sazizeh@hct.ac.ae (S.A.); h00443968@hct.ac.ae (S.R.A.); h00414698@hct.ac.ae (M.A.); h00390704@hct.ac.ae (S.S.A.); h00415698@hct.ac.ae (S.A.A.); h00351797@hct.ac.ae (H.A.); h00351857@hct.ac.ae (A.A.); 4 College of Nursing, Dubai Medical University, Dubai P.O. Box 20170, United Arab Emirates; dr.sarah.s@dmu.ae; 5 Nursing Program, Fatima College of Health Science, Al-Ain Campus, Abu Dhabi P.O. Box 24162, United Arab Emirates; hana.ghunaim@actvet.gov.ae

**Keywords:** physical activity, occupational physical activity, total physical activity, sleep quality, United Arab Emirates, public health

## Abstract

**Highlights:**

**Public health relevance—How does this work relate to a public health issue?**

- Sleep quality and physical inactivity are major public health concerns linked to chronic disease, reduced productivity, and poorer quality of life.
- This study addresses how occupational physical activity and total physical activity relate differently to sleep quality among working adults in the UAE.

**Public health significance—Why is this work of significance to public health?**

- The findings show that total physical activity is associated with better sleep quality at the bivariate level. Among occupational activity domains, only heavy labor showed an independent association with sleep quality after adjustment; because the occupational domain block did not significantly improve overall model fit, this finding should be interpreted as exploratory rather than a confirmed independent effect.
- The study contributes region-specific evidence from the UAE and supports the growing literature on the “physical activity paradox” in relation to sleep health.

**Public health implications—What are the key implications or messages for practitioners, policy makers and/or researchers in public health?**

- Given the small effect sizes and cross-sectional design, these findings are preliminary but tentatively suggest that public health and workplace interventions should not assume that all movement or occupational exertion confers equal sleep-health benefits.
- Future sleep and physical activity strategies should account for activity domain and occupational context when designing health promotion policies and interventions.

**Abstract:**

Physical activity is an important determinant of sleep health; however, its relationship with sleep quality may differ depending on the domain in which it occurs. This study examined the differential associations of occupational physical activity (OPA) and total physical activity (TPA) with sleep quality among adults in the United Arab Emirates. Data were collected from 1310 adults (February–April 2024) using the International Physical Activity Questionnaire, the Occupational Sitting and Physical Activity Questionnaire, and the Sleep Quality Scale, in an analytical cross-sectional design. Higher TPA was associated with better sleep quality at the bivariate level (r = −0.104, *p* < 0.001) but not independently after adjustment (β = −0.042, *p* = 0.246); this null adjusted finding was sensitive to how skewness in the activity data was handled. Occupational sitting was associated with poorer sleep quality only at the bivariate level, whereas occupational heavy labor remained independently associated with better sleep quality after adjustment (β = −0.080, *p* = 0.022). Standing, walking, and the TPA × sitting interaction were not significant. These findings indicate that the physical activity–sleep quality association depends on domain and support promoting overall activity alongside attention to occupational demands; however, given the small effect sizes, cross-sectional design, and unmeasured confounders, these implications should be regarded as preliminary rather than a basis for policy recommendations.

## 1. Introduction

Physical activity is a fundamental determinant of health, influencing a wide range of physiological and psychological outcomes, including sleep quality [1]. Adequate sleep is essential for cognitive functioning, emotional regulation, and overall well-being, whereas poor sleep has been linked to increased risks of cardiovascular disease, obesity, and mental health disorders [2,3,4]. In recent decades, rapid urbanization, technological advancement, and changes in occupational structures have substantially altered physical activity patterns worldwide, particularly in high-income countries such as the United Arab Emirates (UAE) [5]. These transitions have contributed to increasingly sedentary lifestyles, raising concerns regarding their impact on sleep health and overall well-being.

Physical activity research distinguishes between comprehensive measures of total activity, which aggregate energy expenditure across occupational, transport, household, and leisure domains, and domain-specific measures that isolate activity occurring in a single context. This distinction carries meaningful implications for health research, since different domains of activity may relate to outcomes such as sleep quality in different ways.

In the broader literature, physical activity is commonly categorized into different domains, most notably leisure-time physical activity (LPA) and occupational physical activity (OPA). LPA refers to voluntary activities performed during free time, such as exercise, sports, and recreational movement, and is consistently associated with positive health outcomes, including improved sleep quality [6,7,8]. In contrast, OPA includes physical activities performed as part of occupational tasks, which are often characterized by prolonged duration, low autonomy, and limited recovery opportunities [9]. Modern work environments in the UAE and similar contexts are increasingly dominated by sedentary occupations, particularly in administrative and service sectors, resulting in prolonged sitting time and reduced occupational energy expenditure [10].

Emerging evidence from studies using domain-specific activity measures suggests that the health effects of physical activity differ depending on its context, a phenomenon referred to as the “physical activity paradox” [11]. While leisure-time physical activity has been consistently linked to improved cardiovascular and mental health outcomes, occupational physical activity does not appear to provide similar benefits and may even be associated with adverse health effects [11,12]. These differences are attributed to variations in intensity, duration, recovery, and the voluntary versus obligatory nature of the activity. In the context of sleep, these mechanisms are particularly relevant. Leisure-time physical activity is believed to enhance sleep through physiological and psychological pathways, including thermoregulation, circadian rhythm stabilization, and reductions in stress and anxiety [13,14]. Conversely, occupational physical activity, especially when characterized by prolonged standing, repetitive tasks, or physical strain, may contribute to fatigue without adequate recovery, potentially impairing sleep quality [15].

Empirical studies using domain-specific measures of leisure-time activity further support these differential effects. Leisure-time physical activity has been associated with improved sleep duration, sleep efficiency, and reduced insomnia symptoms [13,14]. In contrast, occupational physical activity has shown inconsistent associations with sleep, with some studies reporting increased insomnia symptoms and non-restorative sleep among individuals engaged in physically demanding work [15]. A recent large-scale longitudinal study (N = 62,528) similarly found that higher self-reported occupational physical activity prospectively predicted a greater risk of sleep disturbances over a 5-year follow-up period, reinforcing that occupational and leisure-time activity can have divergent long-term associations with sleep [16]. Additionally, occupational sedentary behavior, particularly prolonged sitting, has been linked to poorer sleep quality and increased sleep disturbances [17]. These findings highlight the importance of distinguishing between domains of physical activity when examining their impact on sleep health.

Despite growing international evidence, limited research has examined these relationships within Middle Eastern populations, particularly in the UAE. Unique contextual factors, including climate conditions, occupational structures, and lifestyle behaviors, may influence both physical activity patterns and sleep outcomes [5]. Furthermore, few studies have simultaneously examined both occupational and domain-specific total physical activity within the same analytical model, limiting the ability to understand their relative and independent contributions to sleep quality.

It should be noted that total physical activity in the present study was operationalized using the IPAQ-SF, a widely used instrument that captures physical activity across multiple life domains, including occupational, transport, household, and leisure contexts, rather than isolating leisure-time behavior specifically [18]. Although conceptually related to leisure-time physical activity as discussed above, this measure should not be interpreted as a domain-pure indicator of leisure activity.

From a theoretical perspective, this study is grounded in the physical activity paradox framework and recovery theory, which suggest that health benefits of physical activity depend not only on energy expenditure but also on the context, autonomy, and opportunity for recovery [11,12]. While leisure-time physical activity promotes recovery and restoration, occupational physical activity may impose sustained physiological strain without sufficient recovery, leading to differential effects on sleep.

Therefore, the present study aims to examine the differential associations between occupational physical activity, total physical activity, and sleep quality among adults in the United Arab Emirates. The study addresses the following research question: Is there a measurable difference in the association of occupational physical activity and total physical activity with sleep quality among adults in the UAE? Based on the existing literature and theoretical framework, the following hypotheses were formulated: H1: Higher levels of total physical activity are associated with better sleep quality (lower SQS scores). H2: Higher occupational sitting time is associated with poorer sleep quality. H3: Different domains of occupational physical activity (sitting, standing, walking, and heavy labor) demonstrate differential associations with sleep quality. H4: The associations between physical activity domains and sleep quality remain significant after adjusting for demographic variables.

## 2. Materials and Methods

### 2.1. Study Design and Setting

This study employed an analytical cross-sectional design to examine the associations between occupational physical activity, total physical activity, and sleep quality among adults in the United Arab Emirates (UAE). Data were collected between February and April 2024 across all seven Emirates, including Abu Dhabi, Dubai, Sharjah, Ajman, Umm Al Quwain, Ras Al Khaimah, and Fujairah. The UAE provides a unique context characterized by rapid urbanization, diverse occupational structures, and varying lifestyle behaviors, making it an appropriate setting to investigate the relationship between physical activity domains and sleep outcomes.

### 2.2. Participants and Sampling

The target population consisted of adults aged 18 years and above residing in the UAE. Participants were eligible for inclusion if they were employed (either full-time or part-time), able to read and understand Arabic or English, and willing to provide informed consent. Individuals with conditions that limited mobility or participation in physical activity were excluded from the study. Participants were recruited using a convenience sampling approach, both through in-person recruitment in public areas such as shopping malls and parks and via online distribution of the survey link. Although convenience sampling may limit generalizability, it was appropriate given the large and diverse population targeted in this study [19].

The sample size for this study was estimated a priori using GPower version 3.1.9.7. Given the study’s hypotheses examining differential associations of occupational and total physical activity domains with sleep quality while adjusting for demographic covariates, a multiple linear regression analysis was assumed as the primary statistical test. With a conservative small effect size (f^2^* = 0.02), a two-tailed significance level (α = 0.05), high statistical power (1-β = 0.95), and up to 10 predictor variables (including total physical activity, occupational sitting, standing, walking, heavy labor, and five demographic covariates), the minimum required sample size was calculated to be 656 participants. To account for an anticipated dropout rate of 10%, the sample size was inflated accordingly, resulting in a final recruitment target of 729 adults aged 18 years and above residing in the United Arab Emirates. A total of 1310 participants met the inclusion criteria and were included in the final analysis. Although this a priori calculation used a simplified set of five conceptual demographic covariates for tractability, the final regression models included a fully dummy-coded set of categorical demographic predictors; the achieved sample exceeds both the calculated minimum and the inflated recruitment target by a substantial margin and therefore provides more than adequate power under the fully specified model as well.

### 2.3. Data Collection Procedure

Data were collected using a structured, self-administered questionnaire developed and hosted on Microsoft Forms and made available in both English and Arabic to enhance accessibility and participation among the diverse population in the UAE. The original instruments were administered in English and translated into Arabic by a bilingual research team to facilitate comprehension among Arabic-speaking participants. Given that the present study was not designed as a formal validation or cultural adaptation study, a standardized forward–backward translation procedure was not implemented. Instead, the translation process focused on ensuring clarity, conceptual equivalence, and linguistic appropriateness. The translated version was reviewed by bilingual researchers with expertise in health sciences to ensure consistency with the original meaning of the items. Of the 1310 participants, 757 (57.8%) completed the English-language version and 553 (42.2%) completed the Arabic-language version of the questionnaire.

Prior to the main data collection, the questionnaire was pilot-tested on approximately 10% of the target sample to assess clarity, readability, and cultural appropriateness. Feedback from the pilot phase indicated that the items were well understood, and no major modifications were required. Both language versions were administered concurrently during data collection. Participants were approached in person in public settings or via the online survey link and completed the questionnaire voluntarily at their convenience (informed consent procedures are described in Section 2.5). The estimated completion time ranged between 5 and 10 min.

### 2.4. Measures

All study variables were assessed using standardized self-report instruments, originally developed in English and translated into Arabic as described in Section 2.3.

*Demographic Characteristics:* Demographic data were collected using a structured questionnaire developed for this study. Variables included age, gender, nationality, marital status, education level, monthly income, and employment status. These variables were treated as categorical variables and were used as covariates in the analysis.

*Total Physical Activity:* Total physical activity was assessed using the International Physical Activity Questionnaire–Short Form (IPAQ-SF), a widely validated instrument that captures self-reported physical activity across multiple life domains, including occupational, transportation, household/yard, and leisure-time activity over the previous seven days [18]. Consistent with standard IPAQ-SF administration, participants were not instructed to restrict their responses to any single domain; therefore, the resulting score reflects overall physical activity rather than leisure-time activity specifically, and may include some activity also captured by the OSPAQ occupational measures. The IPAQ captures the frequency and duration of vigorous, moderate, and walking activities. Total physical activity was calculated in metabolic equivalent (MET) minutes per week using standard scoring procedures. Vigorous activities were assigned a value of 8.0 METs, moderate activities 4.0 METs, and walking 3.3 METs. Total physical activity was computed by summing the MET-minutes for all activity domains. Based on established IPAQ scoring guidelines, participants were categorized into low, moderate, or high physical activity levels [18].

*Occupational Physical Activity:* Occupational physical activity was measured using the Occupational Sitting and Physical Activity Questionnaire (OSPAQ) [20]. The OSPAQ assesses the proportion of time spent sitting, standing, walking, and engaging in physically demanding tasks during a typical workday over the past seven days. Participants reported the number of hours worked and the number of days worked during the previous week, as well as the percentage of time spent in each activity domain. The duration of time spent in each activity (expressed in minutes per day) was calculated by multiplying the reported percentage of time in each activity by the average number of hours worked per day. This approach provides a detailed estimate of occupational physical activity across multiple domains.

Prior to analysis, the four reported activity percentages were checked for internal consistency. Across the full sample (N = 1310), participants reported working an average of 7.66 h per day (SD = 2.54, range 0.8–24.0). The four OSPAQ percentages (sitting, standing, walking, and heavy labor) summed to exactly 100% for 1309 of 1310 participants (99.9%). One participant had a missing value for the heavy labor percentage; because the remaining three domains for this participant already summed to 100%, the missing value was set to 0%, consistent with a fixed workday time budget. Minutes per day for each domain were calculated as the reported percentage multiplied by the participant’s own reported daily working hours, such that the four domain-specific times summed exactly to each participant’s total reported workday.

*Sleep Quality:* Sleep quality was assessed using the Sleep Quality Scale (SQS) developed by Yi et al. [21]. The SQS is a 28-item self-report instrument designed to assess multiple dimensions of sleep quality, including sleep initiation, maintenance, restoration, daytime dysfunction, and satisfaction. Each item is rated on a four-point Likert scale ranging from 0 (“rarely”) to 3 (“almost always”). Seven positively worded items assessing restoration after sleep and satisfaction with sleep (items 2, 8, 13, 16, 18, 20, and 27—e.g., “I feel refreshed after sleep,” “I am satisfied with my sleep”) were reverse-scored prior to analysis (recoded as 3 minus the original response) so that higher values consistently indicated poorer sleep across all 28 items. The resulting total is referred to throughout as the corrected SQS score, to distinguish it from an uncorrected sum of the raw item responses; it was computed by summing all 28 items after this reverse-scoring. Total scores range from 0 to 84, with higher scores indicating poorer sleep quality. In the present sample, the SQS demonstrated good internal consistency (Cronbach’s α = 0.83); reliability was acceptable to good in both the English-language (α = 0.79) and Arabic-language (α = 0.86) versions of the questionnaire.

### 2.5. Ethical Considerations

The study was conducted in accordance with the ethical principles outlined in the Declaration of Helsinki for research involving human participants. Ethical approval was obtained from the Research Ethics Integrity Committee (REIC) at the Higher Colleges of Technology (REIC2024-CAP06) prior to data collection. Participation in the study was entirely voluntary. All participants were provided with a detailed electronic informed consent form prior to accessing the questionnaire. The consent form clearly outlined the purpose of the study, procedures involved, expected duration of participation, potential risks and benefits, and the rights of participants, including the right to withdraw at any time without penalty. Participants were required to indicate their consent electronically before proceeding to the survey. No personally identifiable information, such as names, contact details, or identification numbers, was collected. Anonymity was ensured through the use of non-identifiable data collection methods. Data confidentiality was strictly maintained, and all collected data were stored securely on password-protected systems accessible only to the research team. The study posed minimal risk to participants, as it involved self-reported measures of physical activity and sleep. No financial or material incentives were provided for participation. All procedures adhered to institutional and international ethical standards for human subjects research.

### 2.6. Statistical Analysis

All statistical analyses were conducted using IBM SPSS Statistics (Version 29; IBM Corp., Armonk, NY, USA). Descriptive statistics were used to summarize the study variables, with categorical variables presented as frequencies and percentages, and continuous variables reported as means and standard deviations. Prior to inferential analyses, data were screened for completeness, outliers, and distributional assumptions. No missing data were present for any of the study variables in the final analytic sample (N = 1310); all participants provided complete responses on all IPAQ-SF, OSPAQ, SQS, and demographic items, and no imputation or case deletion was therefore required. The normality of continuous variables was assessed using skewness and kurtosis statistics, and multicollinearity among predictors was evaluated using variance inflation factors.

Pearson correlation analysis was performed to examine the bivariate associations between total physical activity, occupational physical activity domains, and sleep quality. To assess the independent associations of total and occupational physical activity with sleep quality, multiple linear regression analysis was conducted using the total Sleep Quality Scale (SQS) score as the dependent variable. Total physical activity (expressed per 1000 MET-minutes/week) and occupational physical activity domains (each expressed per hour per day spent sitting, standing, walking, and performing heavy labor) were entered as independent variables. This rescaling was applied because a one-unit change on the original scale (1 MET-minute/week or 1 min/day) would not be clinically meaningful; the transformation improves the interpretability of the resulting regression coefficients.

Following the official IPAQ data-cleaning guidelines [22], which recommend truncating any single reported Walking, Moderate, or Vigorous activity duration exceeding 180 min/day to normalize the distribution of activity data prior to analyses based on means, this truncation rule was applied to total physical activity and used as the primary specification in all analyses reported below. As a sensitivity check, given the well-documented skewness of IPAQ data, supplementary analyses using the original, untruncated total physical activity variable, together with Spearman correlations, were also conducted and are reported in Section 3.7.

A hierarchical regression approach was used to examine the incremental contribution of each set of predictors. In the first model, demographic covariates were entered. In the second model, total physical activity was added. In the third model, occupational physical activity variables were entered. In the final model, an interaction term between total physical activity and occupational sitting time was included to examine whether total physical activity moderated the association between occupational sitting and sleep quality. Prior to creating the interaction term, the relevant continuous predictors were mean-centered to reduce multicollinearity and improve interpretability. This interaction was not among the four primary hypotheses formulated a priori, and should therefore be regarded as an exploratory, secondary analysis rather than a prespecified confirmatory test. Occupational sitting was selected as the moderator of interest, rather than occupational standing, walking, or heavy labor, because the existing literature on joint or combined physical activity exposures has focused specifically on the combination of high occupational sedentary behavior with low leisure-time or overall physical activity, rather than on interactions involving other occupational domains [23]. No other physical activity × occupational domain interactions were tested; restricting the exploratory interaction analysis to the single combination with the clearest a priori theoretical basis was a deliberate choice intended to limit multiple exploratory testing.

Statistical significance was set at *p* < 0.05. Effect estimates were reported using unstandardized coefficients, standardized beta coefficients, 95% confidence intervals, and coefficients of determination (R^2^ and ΔR^2^), where appropriate. Effect sizes were interpreted using Cohen’s [24] conventions, whereby standardized β coefficients of approximately 0.10, 0.30, and 0.50 correspond to small, medium, and large effects, respectively, and Cohen’s f^2^ values of 0.02, 0.15, and 0.35 correspond to small, medium, and large effects for R^2^ and ΔR^2^. Because occupational sitting, standing, walking, and heavy labor together constitute a fixed workday time budget (i.e., compositional data that sum to a constant), entering all four domains simultaneously as independent predictors can complicate the interpretation of individual coefficients, since an increase in one domain necessarily implies a decrease in one or more others [25,26]. To address this, a supplementary isotemporal substitution model [27] was estimated in which occupational sitting was treated as the implicit reference category; this model included total occupational time and the remaining three domains (standing, walking, and heavy labor), such that each coefficient estimates the association of reallocating a given amount of time from sitting to that domain, holding total occupational time and total physical activity constant. Unlike the primary regression models described above, this isotemporal model retained the original minute-based units for the occupational domains (rather than the hour-rescaled units) so that coefficients could be interpreted directly as the effect of reallocating a given number of minutes between domains.

## 3. Results

Prior to inferential analyses, the assumptions for Pearson correlation and multiple linear regression were assessed for the primary (IPAQ-truncated) specification. Continuous variables were screened for outliers and distributional characteristics. The occupational activity domains (sitting, standing, walking, heavy labor) remained substantially right-skewed even after truncation (skewness ranging from 1.28 to 1.74), whereas the truncated total physical activity variable showed only mild skewness (skewness = 0.36); despite this, inspection of the regression residuals for the primary model indicated approximate normality. Multicollinearity was not observed, with variance inflation factor values within acceptable limits (all VIFs < 3) despite the compositional nature of the occupational domains. Diagnostic assessment of the primary (truncated) model indicated no influential outliers that materially affected model estimates; by contrast, Section 3.7 shows that a subset of extreme, untruncated activity values did materially influence the total physical activity finding specifically, which is the basis for reporting the truncated specification as primary. With these considerations in mind, the assumptions of linear regression were considered sufficiently met to proceed with the planned analyses.

### 3.1. Participant Characteristics

A total of 1310 adults were included in the final analysis. The largest age group was 30–35 years (28.4%), followed by those aged 24–29 years (24.2%), 36–41 years (18.9%), 18–23 years (17.2%), and participants aged 42 years or older (11.3%). The sample demonstrated a nearly balanced gender distribution (51.5% female, 48.5% male), and the majority were Emirati (68.6%). Regarding education, a bachelor’s degree was the most frequently reported qualification (42.4%), followed by a diploma (17.0%), high school (16.5%), master’s degree (16.3%), and doctoral degree (7.8%). More than half of the participants were married (55.9%), while 34.0% were single. The most commonly reported monthly income category was 20,001–25,000 AED (21.2%), and the vast majority of respondents were employed full-time (76.9%). Additional sample characteristics are presented in Table 1.

### 3.2. Descriptive Statistics of Study Variables

Descriptive statistics for the main study variables are shown in Table 2. The mean corrected Sleep Quality Scale (SQS) score (see Section 2.4 for scoring and reverse-coded items) was 37.69 (SD = 11.27), with observed scores ranging from 12 to 74, indicating substantial variability in perceived sleep quality across the sample. The mean level of total physical activity was 6878.00 MET-min/week (SD = 5202.31), reflecting a wide range of activity engagement. With respect to occupational activity, participants reported spending an average of approximately 136.87 min/day sitting, 108.49 min/day standing, 111.52 min/day walking, and 102.51 min/day performing heavy labor or physically demanding tasks during a typical workday.

### 3.3. Correlation Analysis

Pearson correlation coefficients among the main study variables are presented in Table 3. Total physical activity demonstrated a small but statistically significant negative correlation with sleep quality scores (r = −0.104, 95% CI [−0.157, −0.050], *p* < 0.001), indicating that higher total physical activity was associated with better sleep quality (lower SQS scores). Occupational sitting time showed a small positive correlation with sleep quality scores (r = 0.063, 95% CI [0.009, 0.117], *p* = 0.023), suggesting that greater sitting time at work was associated with poorer sleep quality. In contrast, occupational standing and walking were not significantly associated with sleep quality (r = 0.006, 95% CI [−0.048, 0.060], *p* = 0.834; r = 0.012, 95% CI [−0.043, 0.066], *p* = 0.675, respectively). Occupational heavy labor demonstrated a small but significant negative correlation with sleep quality (r = −0.111, 95% CI [−0.165, −0.058], *p* < 0.001), indicating that greater engagement in physically demanding work was associated with better sleep quality.

Total physical activity was also correlated with the occupational activity domains themselves (Table 3). Total physical activity showed a moderate-to-strong positive correlation with occupational heavy labor (r = 0.620, *p* < 0.001), sharing approximately 38% of variance with this domain; because IPAQ-SF vigorous-activity items instruct respondents to include occupation-related exertion, this overlap is consistent with the instrument’s design rather than indicating a data anomaly. In contrast, total physical activity showed a weaker correlation with occupational sitting (r = −0.452, *p* < 0.001, ~20% shared variance) and negligible correlations with occupational standing (r = −0.110, *p* < 0.001) and occupational walking (r = −0.029, *p* = 0.302). This pattern indicates that the overlap between total physical activity and occupational activity is concentrated specifically in the heavy labor domain rather than reflecting broad redundancy across all occupational domains; findings involving heavy labor specifically should therefore be interpreted with this shared variance in mind, whereas total physical activity and the sitting, standing, and walking domains remain meaningfully distinct.

### 3.4. Hierarchical Multiple Linear Regression Analysis

A hierarchical multiple linear regression analysis was conducted to examine the independent associations of total and occupational physical activity with sleep quality. The corrected total SQS score was entered as the dependent variable. Demographic variables were entered in Model 1, total physical activity was added in Model 2, occupational physical activity domains were added in Model 3, and the interaction term between total physical activity and occupational sitting time was entered in Model 4. The full model is presented in Table 4. In Model 1, demographic variables explained 3.5% of the variance in sleep quality (R^2^ = 0.035, *p* < 0.001). In Model 2, the addition of total physical activity significantly improved the model, increasing the explained variance to 4.2% (ΔR^2^ = 0.007, *p* = 0.003). Total physical activity (per 1000 MET-min/week) emerged as a significant predictor of sleep quality (b = −0.194, β = −0.089, *p* = 0.003), indicating that greater total physical activity was associated with lower sleep disturbance.

In Model 3, the inclusion of occupational activity variables produced a modest increase in explained variance to 4.6%, although the overall change in explained variance was not statistically significant (ΔR^2^ = 0.004, *p* = 0.215). In this model, total physical activity (per 1000 MET-min/week) was no longer an independent predictor of sleep quality (b = −0.090, β = −0.042, *p* = 0.246). Among the occupational activity domains (each expressed per hour/day), only heavy labor was significantly associated with sleep quality (b = −0.568, β = −0.077, *p* = 0.027), indicating that greater time spent in physically demanding occupational tasks was associated with better sleep quality. Occupational sitting, standing, and walking were not significant predictors in the adjusted model. Because the overall occupational activity block did not significantly improve model fit, and four domain-specific coefficients were examined without adjustment for multiple comparisons, the heavy labor coefficient should be interpreted as an exploratory finding rather than a confirmed independent effect.

In Model 4 (Table 5), the interaction term between total physical activity and occupational sitting time was entered to test whether total physical activity moderated the relationship between occupational sitting and sleep quality. The addition of the interaction term resulted in only a minimal increase in explained variance (R^2^ = 0.049), and the interaction effect did not reach statistical significance, although it approached the conventional threshold (b = 0.072, β = 0.052, *p* = 0.084). This finding indicates that total physical activity did not significantly buffer or modify the association between occupational sitting time and sleep quality in this sample.

Overall, the findings indicate that higher total physical activity is associated with better sleep quality, although this association did not remain statistically significant after adjustment for demographic and occupational variables in the primary model, and the bivariate effect size was, in any case, modest. Among occupational activity domains, only heavy labor showed a significant independent association with sleep quality, while occupational sitting was associated with poorer sleep quality only at the bivariate level. The hypothesized moderation effect of total physical activity on the association between occupational sitting and sleep quality was not supported. In terms of effect size, the full model corresponded to a small effect (Cohen’s f^2^ = 0.051), and the incremental contributions of total physical activity (f^2^ = 0.007), occupational domains (f^2^ = 0.005), and the interaction term (f^2^ = 0.002) were all below Cohen’s conventional threshold for a small effect (f^2^ = 0.02); similarly, the standardized β coefficients for total physical activity (β = −0.089 in Model 2) and occupational heavy labor (β = −0.080 in the final model) both correspond to small effects by conventional standards. These effect sizes should be interpreted in light of the cross-sectional design and the large sample size, which affords statistical power to detect associations that are modest in practical magnitude.

### 3.5. Sensitivity Analysis by Questionnaire Language

To evaluate whether questionnaire language influenced the observed associations, a series of sensitivity analyses was conducted. Bivariate correlations between total physical activity and sleep quality did not differ significantly between the English- and Arabic-language respondents (English: r = −0.144; Arabic: r = −0.050; Fisher’s z = −1.70, *p* = 0.089), whereas correlations between occupational sitting and sleep quality differed significantly by language (English: r = −0.028; Arabic: r = 0.159; z = −3.36, *p* = 0.001). Correlations for occupational standing, walking, and heavy labor did not differ significantly by language (all *p* > 0.19). To formally test whether questionnaire language moderated the associations observed in the adjusted model, physical activity × language interaction terms (total physical activity, occupational sitting, and occupational heavy labor, each × language) were added jointly to the final hierarchical regression model. This produced a statistically significant improvement in model fit (ΔR^2^ = 0.021, F(3,1281) = 9.62, *p* < 0.001), indicating that questionnaire language moderates the joint pattern of associations between physical activity and sleep quality even though the simple bivariate association for total physical activity alone did not differ significantly by language group. These findings are considered further in Section 4.6.

### 3.6. Isotemporal Substitution Analysis

To evaluate whether the compositional structure of the occupational activity domains affected the interpretation of the primary regression results, a supplementary isotemporal substitution model was estimated (Section 2.6). This model explained the same proportion of variance in sleep quality (R^2^ = 0.046) as the primary domain-specific model (R^2^ = 0.046; Table 4), as expected given that the two specifications are algebraically equivalent reparameterizations of the same predictors. Reallocating occupational sitting time to heavy labor was significantly associated with better sleep quality (b = −0.011 per minute reallocated, equivalent to −0.34 points per 30 min/day, SE = 0.005, *p* = 0.025), consistent with the protective association observed for heavy labor in the primary model. Reallocating sitting time to standing (b = −0.0004, *p* = 0.945) or walking (b = 0.002, *p* = 0.779) was not significantly associated with sleep quality, nor was total occupational time itself (b = 0.002, *p* = 0.592). These results are directionally consistent with the primary model’s finding regarding occupational heavy labor across this alternative compositional specification; given that the overall occupational activity block did not significantly improve model fit in the primary model and that four domain-specific coefficients were examined without correction for multiple comparisons, this finding should nonetheless be interpreted as exploratory rather than robust.

### 3.7. Sensitivity Analysis for Skewness and Data Cleaning

Total physical activity showed a right-skewed distribution prior to data cleaning (skewness = 1.11, kurtosis = 0.93; median = 7447.5, IQR [1980.0, 13,560.0] MET-min/week), consistent with previously reported patterns for IPAQ-derived data; for comparison, IPAQ classification guidelines consider individuals exceeding 3000 MET-min/week to already be highly active, underscoring that the untruncated mean and upper values in this sample were driven by a subset of extreme responses rather than being representative of the median participant. Following the official IPAQ data-cleaning guidelines [22], which recommend truncating any single reported activity duration exceeding 180 min/day, this truncation was applied to total physical activity in all primary analyses reported above. In total, 643 of 1310 participants (49.1%) had at least one activity duration exceeding this threshold and were affected by truncation. Applying this rule reduced the skewness of total physical activity to 0.36 (kurtosis = −1.01) and its median to 6468.0 (IQR [1907.3, 11,016.0]) MET-min/week, and reduced its mean from 9109 to 6878 MET-min/week, consistent with the guidelines’ stated purpose of normalizing the distribution of activity data prior to analyses based on means, such as the regression models reported here.

To further evaluate the robustness of the primary findings, Spearman correlations were computed alongside the Pearson correlations reported in Table 3. These were similar in magnitude to, and slightly stronger than, the Pearson correlations for both total physical activity (Pearson r = −0.104 vs. Spearman ρ = −0.153, both *p* < 0.001) and occupational heavy labor (Pearson r = −0.111 vs. Spearman ρ = −0.143, both *p* < 0.001), supporting the robustness of the reported bivariate associations to the underlying skew in the activity data.

As a sensitivity check, the primary regression models were also estimated using the original, untruncated total physical activity variable. The bivariate correlation with sleep quality was similar in magnitude under this specification (r = −0.115, *p* < 0.001), and the independent association in the fully adjusted model was statistically significant (b = −0.087 per 1000 MET-min/week, *p* = 0.048), compared with a non-significant association using the truncated variable (b = −0.077, *p* = 0.327; Table 5). This discrepancy indicates that the significant adjusted association observed with untruncated data was disproportionately influenced by a subset of participants reporting implausibly high activity durations (up to 43,836 MET-min/week, equivalent to several hours of vigorous activity on every day of the week); once these values were truncated in accordance with standard IPAQ guidance, the independent association for total physical activity was no longer significant. We report the truncated specification as primary because it follows established, published data-cleaning procedures for IPAQ data (Section 2.6) and because the untruncated values imply activity levels that are difficult to reconcile with sustained weekly patterns. The occupational heavy labor finding was materially unaffected by this choice and was, if anything, slightly stronger under truncation (Table 5).

### 3.8. Sex-Stratified and Interaction Analysis

Given well-established sex differences in sleep quality, physical activity patterns, and occupational exposure, sex-stratified bivariate correlations and a formal test of physical activity × sex interactions were conducted. Bivariate correlations between total physical activity and sleep quality did not differ significantly by sex (women: r = −0.141, *p* < 0.001; men: r = −0.065, *p* = 0.104; Fisher’s z = −1.40, *p* = 0.162), nor did correlations between occupational heavy labor and sleep quality (women: r = −0.127, *p* = 0.001; men: r = −0.097, *p* = 0.015; z = −0.55, *p* = 0.581). Correlations between occupational sitting and sleep quality differed by sex at a level approaching significance (women: r = 0.010, *p* = 0.803; men: r = 0.115, *p* = 0.004; z = −1.92, *p* = 0.055), suggesting a stronger positive association between sitting and poorer sleep quality among men than women.

A joint test of total physical activity × sex, occupational heavy labor × sex, and occupational sitting × sex interaction terms, added to the fully adjusted regression model, did not significantly improve model fit overall (ΔR^2^ = 0.004, F(3,1282) = 1.97, *p* = 0.117). Examined individually, the total physical activity × sex (b = 0.158, *p* = 0.297) and heavy labor × sex (b = −0.148, *p* = 0.619) interactions were not significant, but the occupational sitting × sex interaction was significant (b = 0.582, *p* = 0.026), consistent with the bivariate finding above. When the fully adjusted model was estimated separately within each sex, occupational heavy labor was directionally protective in both women (b = −0.217, *p* = 0.232) and men (b = −0.396, *p* = 0.130) but did not reach statistical significance in either stratum, likely reflecting the reduced statistical power of analyzing approximately half the sample in each group. Overall, these results provide no strong evidence that the total physical activity or occupational heavy labor associations with sleep quality differ meaningfully by sex, although occupational sitting may be more strongly associated with poorer sleep quality among men; this sex-specific pattern for sitting warrants replication in future, adequately powered studies.

## 4. Discussion

This analytical cross-sectional study examined the differential associations of total and occupational physical activity with sleep quality among adults in the United Arab Emirates. Three findings are particularly noteworthy. First, higher total physical activity was associated with better sleep quality, although this association did not remain statistically significant once demographic and occupational variables were adjusted for in the primary (IPAQ-truncated) model, and the bivariate effect size was, in any case, modest. Second, occupational physical activity was not uniform in its relationship with sleep. Of the occupational domains examined, only heavy labor remained independently associated with sleep quality in the adjusted model, whereas sitting was associated with poorer sleep only at the bivariate level, and standing and walking were not significant predictors. Third, the hypothesized interaction between total physical activity and occupational sitting was not supported, suggesting that the beneficial association of total physical activity with sleep quality did not significantly vary according to the level of occupational sitting in this sample.

Taken together, these findings support the view that physical activity should not be treated as a single undifferentiated construct when sleep outcomes are examined. Rather, the context, structure, and likely physiological meaning of physical activity appear to matter. This overall pattern is consistent with the broader literature suggesting that leisure-time physical activity and occupational physical activity may have different, and sometimes opposite, relationships with health outcomes [28,29,30,31].

### 4.1. Total Physical Activity and Sleep Quality

The finding that total physical activity was associated with better sleep quality at the bivariate level is broadly consistent with evidence showing that regular physical activity improves subjective sleep outcomes, including sleep quality, sleep latency, and daytime functioning. Meta-analyses and systematic reviews have shown that both acute and regular exercise are associated with small-to-moderate improvements in sleep, particularly in subjective sleep quality measures [32,33,34]. In the present study, however, this association did not remain statistically significant once demographic and occupational factors were considered in the primary (IPAQ-truncated) model, suggesting that some of the crude bivariate association may be explained by these covariates, or by a subset of extreme reported activity values (Section 3.7), rather than reflecting a robust independent relationship with sleep in this population.

Several mechanisms may plausibly explain the bivariate association observed between total physical activity and sleep quality. Because the IPAQ-SF does not isolate leisure-time activity from occupational, transport, and household exertion, this association should not be interpreted as reflecting leisure-time activity specifically; indeed, total physical activity shared a substantial proportion of variance with occupational heavy labor in this sample (r = 0.620, r^2^ = 0.384; Table 3), indicating that occupational exertion contributes meaningfully to the total physical activity score. To the extent that some portion of total physical activity nonetheless reflects voluntary, self-paced movement occurring outside structured work demands, several mechanisms may be relevant: such activity is more likely to occur under conditions of personal control and adequate recovery, and exercise has also been linked to improved thermoregulation, circadian rhythm synchronization, reduced arousal, and better mood regulation, all of which may facilitate sleep [32,33]. However, given the substantial shared variance between total physical activity and occupational heavy labor specifically, part of the observed association may also reflect the health-related correlates of vigorous occupational exertion itself, rather than leisure engagement exclusively.

It should be emphasized explicitly that the final model explained only R^2^ = 0.049 of the variance in sleep quality, meaning that approximately 95% of the variance remains unexplained by the physical activity variables and demographic covariates included here. This is a critical result in its own right, and we caution against emphasizing the statistical significance of individual associations (e.g., for occupational heavy labor) without equal attention to this limited predictive value; the associations reported in this study, while statistically detectable given the large sample size, account for only a small fraction of what determines sleep quality in this population. At the same time, the effect size in the present study was modest. This is not surprising. Sleep quality is a multidetermined outcome influenced not only by physical activity but also by work demands, mental health, family responsibilities, screen exposure, caffeine use, and broader lifestyle factors [35]. Therefore, the observed modest effect should not be interpreted as trivial; rather, it likely reflects the realistic contribution of one important behavioral determinant within a larger sleep-health ecosystem. This magnitude of explained variance is also consistent with comparable studies in this literature: a recent cross-sectional study of a similarly sized sample of university students using IPAQ-based total physical activity reported an almost identical explained variance in sleep quality (R^2^ = 0.031) [36], and meta-analytic evidence indicates that physical activity is reliably, but modestly, associated with sleep outcomes across studies [13]. The present model was designed to identify and characterize associations between specific activity domains and sleep quality, not to serve as a predictive or explanatory model of sleep quality as a whole; the significant independent association observed for occupational heavy labor should therefore be interpreted as one exploratory, partial contributor within a much larger set of determinants, rather than as a definitive or generalizable effect.

It is also worth noting that total physical activity was more strongly correlated with occupational heavy labor (r = 0.620, *p* < 0.001) than with any other study variable (Table 3). This pattern suggests substantial overlap between the IPAQ-SF-based total physical activity measure and occupationally demanding exertion, and may help explain both the bivariate association between total physical activity and sleep quality and why this association did not persist independently of occupational heavy labor in the adjusted model.

### 4.2. Occupational Physical Activity and the Physical Activity Paradox

The occupational findings were more complex, and this complexity is itself meaningful. The non-significant adjusted associations for occupational sitting, standing, and walking, together with the significant inverse association for heavy labor, suggest that occupational activity is heterogeneous rather than uniformly harmful or beneficial. This is an important point, because the literature increasingly cautions against assuming that all movement at work confers the same health benefits as exercise performed during leisure time [29,30,37].

The broader “physical activity paradox” framework proposes that occupational physical activity may fail to produce the same health benefits as domain-specific leisure-time physical activity because it is often characterized by prolonged exposure, static loading, insufficient recovery, limited worker autonomy, and elevated physiological strain without corresponding improvements in fitness [30,31]. In relation to sleep, this framework is relevant because work-related physical demands may contribute to fatigue, musculoskeletal discomfort, or physiological stress in ways that do not translate into more restorative sleep. Previous work has shown that occupational physical activity can be associated with insomnia symptoms and poorer sleep outcomes, whereas domain-specific leisure-time physical activity is more consistently associated with better sleep [37].

In the present study, occupational sitting was associated with poorer sleep quality at the bivariate level but lost statistical significance in the adjusted model. This suggests that the crude association between sitting and sleep may be partly explained by confounding factors, or that occupational sitting alone is not a sufficiently strong independent predictor once broader activity patterns are considered. This does not mean that occupational sitting is unimportant. Rather, it suggests that its association with sleep may operate through more proximal behavioral or psychosocial pathways not directly measured in this study, such as prolonged screen exposure, mental fatigue, low job control, or reduced physical recovery after work.

The finding that standing and walking at work were not significantly associated with sleep quality after adjustment is also informative. These activities may not have been of sufficient intensity, duration pattern, or physiological meaning to influence sleep in a measurable way. Unlike structured exercise, occupational standing and walking are often fragmented, task-driven, and embedded within work demands. Their health relevance may therefore depend more on the context in which they occur than on their mere duration.

### 4.3. Interpreting the Heavy Labor Finding as an Exploratory Association

One of the more interesting findings of this study was that greater time spent in heavy labor was independently associated with better sleep quality. This effect emerged despite the overall occupational activity block not significantly improving model fit (ΔR^2^ = 0.004, *p* = 0.215), and four domain-specific coefficients were examined without adjustment for multiple comparisons; accordingly, this finding should be interpreted as an exploratory association rather than a robust or confirmed independent effect. It was, in addition, modest in absolute terms (β = −0.077, Table 5), and its 95% confidence interval approached the null value. At first glance, this finding may also seem inconsistent with the physical activity paradox. Several explanations are plausible, but we emphasize at the outset that no physiological, objective (e.g., accelerometer-based), or occupational classification data were collected in this study, so the mechanistic interpretations offered below are speculative and not directly tested with the available data; they are intended to generate hypotheses for future research rather than to establish causal mechanisms.

First, not all physically demanding work is physiologically equivalent. The heavy labor category in the OSPAQ may capture a subset of workers whose jobs involve higher energy expenditure and lower sitting time but not necessarily the prolonged static strain or adverse psychosocial conditions often emphasized in paradox literature. In other words, the “heavy labor” captured here may reflect meaningful bodily exertion that promotes sleep pressure in at least some occupational contexts. Second, this effect may reflect residual differences in job type, work rhythm, or worker profile that were not fully measured in the present analysis. Third, because occupational activity was self-reported, the heavy labor domain may also reflect perceived exertion or general physical work engagement rather than objectively quantified workload. This important finding aligns with the idea that occupational physical activity should not be treated as a monolithic exposure. Some occupational tasks may indeed be detrimental, particularly when repetitive, prolonged, and poorly recoverable, while others may be less harmful or even beneficial in specific contexts. Reviews in working populations have similarly suggested that the relation between occupational and leisure-time physical activity is not simply additive and may vary according to occupation, work organization, and the broader balance between strain and recovery [2,31,37]. Therefore, the present finding should be interpreted cautiously, but not dismissed. It may indicate that the sleep implications of occupational activity depend on the quality and structure of the activity, not merely its presence. This interpretation is further supported by a supplementary isotemporal substitution analysis (Section 3.6), which confirmed that reallocating time from sitting to heavy labor remained significantly associated with better sleep quality after accounting for the compositional constraints inherent to the occupational activity domains.

### 4.4. Non-Significant Moderation by Total Physical Activity

The interaction between total physical activity and occupational sitting did not reach statistical significance, although it approached the conventional threshold (*p* = 0.084). In practical terms, this means that the beneficial association of total physical activity with sleep quality did not differ significantly across levels of occupational sitting in this sample. This is an important result because it suggests that, although total physical activity was associated with better sleep quality at the bivariate level, it did not clearly buffer the sleep-related impact of occupational sitting in a measurable way.

This finding is not entirely unexpected. Some prior studies have found joint or combined patterns between occupational and leisure activity, but evidence for interaction or buffering effects is inconsistent. For example, previous work has shown that high occupational physical activity combined with low domain-specific leisure-time physical activity may be associated with worse sleep outcomes, yet clear interaction effects are not always observed statistically [23]. The absence of moderation in the present study may therefore reflect the reality that total physical activity and occupational sitting exert largely independent rather than interactive influences on sleep, or that any true interaction is too small to detect using self-reported exposure measures. From a manuscript perspective, this non-significant interaction does not weaken the study. On the contrary, it strengthens the credibility of the analysis by showing that the interaction hypothesis was tested directly rather than assumed. Reporting the null interaction transparently also helps sharpen the interpretation of the main effects.

### 4.5. Relevance to the UAE Context

The present findings are particularly relevant in the UAE, where rapid urbanization, technological dependence, and changing occupational structures have contributed to increasingly sedentary lifestyles and shifting patterns of physical activity. Regional evidence has highlighted the importance of sleep and physical activity for well-being among adults in Abu Dhabi and the wider UAE, while also pointing to the role of work and lifestyle pressures in shaping sleep outcomes [38]. In such a context, distinguishing between overall physical activity and occupational-specific activity is especially important because adults may simultaneously experience sedentary work environments and limited opportunities for restorative physical activity outside work. The current results therefore have practical relevance for workplace health and public health promotion in the UAE. Interventions focused solely on increasing “activity” without considering the domain may miss important distinctions. Encouraging overall physical activity engagement outside of high-intensity occupational demands, reducing prolonged occupational sitting, and promoting healthier work–recovery patterns may tentatively be more effective than undifferentiated activity recommendations.

### 4.6. Strengths and Limitations

This study has several strengths. It included a large adult sample from across the UAE, used established instruments to assess physical activity and sleep, and examined total and occupational physical activity simultaneously within the same analytical framework. The hierarchical regression approach also allowed the relative contribution of total physical activity and occupational domains to be examined after adjustment for demographic factors.

Several limitations should also be acknowledged: First, the cross-sectional design precludes causal inference, and several established determinants of sleep quality were not measured, including caffeine and alcohol intake, smoking, body mass index, chronic disease, mental health (e.g., anxiety, depression), shift work, working hours, job strain, screen exposure, sleep medication use, sleep duration, general psychological stress, family responsibilities, and diagnosed sleep disorders; these represent important sources of residual confounding.

Second, all measures were self-reported, and total physical activity was highly skewed; although IPAQ truncation guidelines [22] were applied as the primary specification and the bivariate association was robust to truncation and to Spearman correlation, the adjusted association became non-significant under this recommended specification while remaining significant with untruncated data, indicating sensitivity to how extreme self-reported values are handled. Relatedly, accelerometer-based validation of the OSPAQ indicates acceptable validity for sitting and standing but poor validity for walking and heavy labor specifically [39]-the domain most central to our key findings. Because the IPAQ-SF was administered without domain-specific instructions, total physical activity also reflects activity across all life domains rather than leisure time specifically, and correlates substantially with occupational heavy labor (r = 0.620, Table 3); a physically demanding worker (e.g., in construction) could plausibly score highly on both measures for the same underlying exertion, so part of the total-physical-activity–sleep association may reflect occupational overlap rather than a distinct leisure-based recovery effect. In addition, the Arabic translation was not formally validated, and sensitivity analyses showed that the physical activity–sleep associations differed by questionnaire language, suggesting some associations may partly reflect measurement differences rather than physical activity itself. This measurement overlap also has direct implications for interpreting the adjusted regression models: because total physical activity and occupational heavy labor are not fully independent exposures, the seemingly independent associations estimated for each in the adjusted models should be interpreted as reflecting entangled rather than cleanly separable underlying constructs. This represents an inherent limitation of the study design rather than an estimation artifact, since no combination of statistical adjustment can fully disentangle two exposures that partly capture the same underlying exertion.

Third, convenience sampling may limit generalizability. Eligibility criteria did not exclude individuals with diagnosed sleep disorders, shift workers, sleep medication users, or pregnant individuals, nor was participants’ specific occupation, job title, or industry recorded beyond OSPAQ time-allocation percentages. This is a notable limitation specifically for interpreting the heavy labor finding: the OSPAQ heavy labor category likely aggregates a physiologically and psychosocially diverse range of occupations (e.g., construction, agriculture, manufacturing, domestic labor), which may differ substantially in exertion intensity, autonomy, safety conditions, and recovery opportunities. Without occupational classification, it is not possible to determine whether the observed association reflects a general effect of physical exertion or is instead driven by specific occupational contexts within the broader heavy labor category. Both gaps may introduce unmeasured confounding and occupational heterogeneity that could not be examined. In addition, recruitment and response information (numbers approached, declined, or excluded) was not recorded, precluding a formal participation flow diagram and evaluation of non-response bias.

Finally, the explained variance was modest, indicating that important determinants of sleep quality were not captured. A supplementary isotemporal substitution analysis yielded directionally consistent results for heavy labor after accounting for the compositional nature of the OSPAQ domains, although a full compositional data analysis (e.g., isometric log-ratio transformation) would be more rigorous [25,26]; this consistency across specifications does not offset the exploratory nature of the finding, given the non-significant occupational block and the absence of correction for multiple domain-specific comparisons. The exploratory sex-stratified and interaction analyses were not among the prespecified hypotheses and were likely underpowered to detect small interaction effects; the borderline sitting × sex interaction should be interpreted cautiously pending replication. Future studies should address these gaps using objectively measured physical activity, validated translations, complete recruitment reporting, and detailed occupational classification.

### 4.7. Implications for Research and Practice

The findings suggest several implications: For practice, the findings tentatively point toward the value of promoting overall physical activity engagement as part of sleep-health strategies for adults in the UAE, and toward workplace approaches that address broader work patterns and recovery opportunities rather than reducing sitting time alone; Section 4.8 discusses important caveats to these practice implications. For research, the results highlight the need for longitudinal and device-based studies that can distinguish more precisely between occupational activity and leisure-time activity specifically (e.g., using the IPAQ long form or accelerometry), quantify intensity and timing, and examine whether specific work sectors or job types modify these associations. Such work would help clarify whether the apparent benefit of heavy labor in the present study reflects a true context-specific effect or residual heterogeneity within occupational roles.

### 4.8. Practical and Public Health Implications

The findings of this study should be regarded as preliminary and hypothesis-generating rather than as a basis for specific policy or programmatic recommendations. The observed associations, while statistically significant for occupational heavy labor, corresponded to small effect sizes, were derived from a cross-sectional design that cannot establish causality or direction of effect, and were obtained without adjustment for several potentially important confounders (e.g., mental health, sleep medication use, caffeine and alcohol intake). Any translation of these findings into workplace or public health practice should therefore await confirmation from longitudinal studies using objectively measured physical activity and a more complete set of covariates. With this caveat, the findings tentatively suggest that programs aimed at improving sleep health should not assume that all physical activity confers equal benefit, and that the specific role of occupational heavy labor and prolonged occupational sitting may warrant further investigation before being incorporated into workplace wellness or public health initiatives in the UAE and similar settings.

## 5. Conclusions

This study contributes to the growing evidence that the relationship between physical activity and sleep quality depends on the domain in which physical activity occurs. Among adults in the UAE, higher levels of total physical activity were associated with better sleep quality, whereas occupational physical activity demonstrated a more heterogeneous pattern. Total physical activity was associated with better sleep quality at the bivariate level but did not remain an independent predictor once standard IPAQ data-cleaning procedures were applied, indicating that this specific finding should be interpreted with caution. In adjusted analyses, occupational heavy labor was the only activity measure that showed an independent association with sleep quality across sensitivity analyses; however, because the overall occupational activity block did not significantly improve model fit and four domain-specific coefficients were examined without correction for multiple comparisons, this finding should be regarded as exploratory rather than robust, while occupational sitting, standing, and walking were not significant predictors. In addition, total physical activity did not significantly moderate the relationship between occupational sitting and sleep quality. These findings support the importance of distinguishing between total physical activity and occupational-specific physical activity when examining sleep health and reinforce the view that physical activity should not be treated as a single, uniform exposure. Given the small effect sizes observed, the cross-sectional design, and the unmeasured confounders discussed in Section 4.6, these findings should be considered preliminary. Any public health or workplace implications are tentative and would require confirmation from longitudinal, objectively measured research; with that caveat, the findings point tentatively toward the value of further examining the promotion of regular physical activity overall, while also considering the broader occupational context in which movement and sedentary behavior occur, rather than as a basis for specific policy or programmatic recommendations at this stage.

Given the cross-sectional design, causal conclusions cannot be drawn. Nevertheless, the findings provide an important foundation for future research in the UAE and similar settings. Longitudinal and objectively measured studies are warranted to clarify the temporal and contextual relationships between different physical activity domains and sleep health and to inform more targeted workplace and population-level health promotion strategies.

## Figures and Tables

**Table 1:** Participant Characteristics (N = 1310).

Variable	n	%
**Age**		
18–23 years	225	17.2
24–29 years	317	24.2
30–35 years	372	28.4
36–41 years	248	18.9
≥42 years	148	11.3
**Gender**		
Female	675	51.5
Male	635	48.5
**Nationality**		
Emirati	899	68.6
Non-Emirati	411	31.4
**Education level**		
High school	216	16.5
Diploma	223	17.0
Bachelor’s degree	555	42.4
Master’s degree	214	16.3
Doctoral degree	102	7.8
**Marital status**		
Single	446	34.0
Married	732	55.9
Divorced	81	6.2
Widowed	51	3.9
**Monthly income**		
0–5000 AED	197	15.0
5001–10,000 AED	145	11.1
10,001–15,000 AED	225	17.2
15,001–20,000 AED	236	18.0
20,001–25,000 AED	278	21.2
>25,000 AED	229	17.5
**Employment status**		
Part-time	302	23.1
Full-time	1008	76.9

**Table 2:** Descriptive Statistics of Main Study Variables.

Variable	n	Mean	SD
Sleep quality score	1310	37.69	11.27
Total physical activity (MET-min/week)	1310	6878.00	5202.31
Occupational sitting (min/day)	1310	136.87	99.32
Occupational standing (min/day)	1310	108.49	61.34
Occupational walking (min/day)	1310	111.52	63.54
Occupational heavy labor (min/day)	1310	102.51	92.27

**Table 3:** Pearson Correlation Matrix among Main Study Variables.

Variable	SQ	TPA	OPA Sitting	OPA Standing	OPA Walking	OPA Heavy Labor
SQ	—	−0.104 **	0.071 *	0.006	0.026	−0.103 **
TPA	−0.104 **	—	−0.452 **	−0.110 **	−0.029	0.620 **
OPA sitting	0.071 *	−0.452 **	—	−0.324 **	−0.438 **	−0.655 **
OPA standing	0.006	−0.110 **	−0.324 **	—	−0.076 **	−0.258 **
OPA walking	0.026	−0.029	−0.438 **	−0.076 **	—	−0.093 **
OPA heavy labor	−0.103 **	0.620 **	−0.655 **	−0.258 **	−0.093 **	—

Note. TPA = total physical activity; OPA = occupational physical activity; SQ = sleep quality. * *p* < 0.05; ** *p* < 0.01. Pearson’s (r) values are shown. Higher SQS scores indicate poorer sleep quality; therefore, negative correlations with SQ indicate an association with better sleep quality. 95% confidence intervals for the correlations between SQ and each physical activity variable are reported in the text (Section 3.3).

**Table 4:** Hierarchical Multiple Linear Regression Predicting Sleep Quality: Model Fit.

Model	Predictors Entered	R^2^	Adjusted R^2^	ΔR^2^	*p* for Block Change
1	Demographic covariates	0.035	0.021	0.035	<0.001
2	+Total physical activity (per 1000 MET-min/wk)	0.042	0.027	0.007	0.003
3	+Occupational domains	0.046	0.029	0.004	0.215
4	+TPA (per 1000) × sitting (per hr) interaction	0.049	0.030	0.002	0.084

**Table 5:** Final Hierarchical Regression Model (Model 4) Coefficients.

Predictor	B	SE	t	*p*	95% CI Lower	95% CI Upper
Intercept	38.310	1.360	28.160	<0.001	35.641	40.979
Age 24–29 years	−1.548	0.925	−1.673	0.095	−3.362	0.267
Age 30–35 years	−1.408	0.923	−1.524	0.128	−3.219	0.404
Age 36–41 years	−0.808	1.022	−0.791	0.429	−2.814	1.197
Age ≥ 42 years	−2.203	1.964	−1.122	0.262	−6.057	1.650
Male	0.392	0.680	0.576	0.565	−0.943	1.727
Non-Emirati	0.739	0.718	1.029	0.304	−0.670	2.148
Diploma	0.761	1.114	0.683	0.495	−1.424	2.947
Bachelor’s degree	2.676	0.954	2.807	0.005	0.806	4.547
Master’s degree	3.640	1.227	2.966	0.003	1.232	6.047
Doctoral degree	−0.404	1.520	−0.266	0.790	−3.387	2.579
Married	0.882	0.765	1.153	0.249	−0.619	2.383
Divorced	−0.691	1.416	−0.488	0.626	−3.469	2.087
Widowed	−3.577	1.720	−2.080	0.038	−6.952	−0.203
Income 5001–10,000 AED	−0.478	1.321	−0.362	0.717	−3.070	2.113
Income 10,001–15,000 AED	−1.245	1.229	−1.013	0.311	−3.656	1.166
Income 15,001–20,000 AED	−2.036	1.237	−1.646	0.100	−4.463	0.391
Income 20,001–25,000 AED	−1.694	1.234	−1.373	0.170	−4.115	0.727
Income > 25,000 AED	−3.254	1.310	−2.484	0.013	−5.824	−0.684
Full-time employment	0.351	0.827	0.424	0.671	−1.272	1.974
Total physical activity (per 1000 MET-min/wk)	−0.077	0.078	−0.981	0.327	−0.230	0.077
Occupational sitting (per hr/day)	0.213	0.217	0.985	0.325	−0.212	0.639
Occupational standing (per hr/day)	0.003	0.320	0.011	0.992	−0.624	0.630
Occupational walking (per hr/day)	0.205	0.312	0.656	0.512	−0.408	0.817
Occupational heavy labor (per hr/day)	−0.586	0.256	−2.287	0.022	−1.088	−0.083
TPA (per 1000) × sitting (per hr) interaction	0.072	0.041	1.727	0.084	−0.010	0.153

Note. Reference categories: age 18–23 years, female, Emirati, high school, single, income 0–5000 AED, and part-time employment. Total physical activity is expressed per 1000 MET-minutes/week; occupational activity domains (sitting, standing, walking, heavy labor) are each expressed per hour (60 min) per day. The dependent variable in this model is the Sleep Quality Scale (SQS) score; higher SQS scores indicate poorer sleep quality, so a negative B coefficient indicates an association with better sleep quality.

## Data Availability

The raw data supporting the conclusions of this article will be made available by the authors on request.

## Abbreviations

The following abbreviations are used in this manuscript:

UAE	United Arab Emirates
TPA	total physical activity
OPA	occupational physical activity
IPAQ-SF	International Physical Activity Questionnaire—Short Form
